# Toward Vision-Based Estrus Monitoring in Chongming White Goats: Behavioral Manifestations and Serum Hormone Correlations

**DOI:** 10.3390/ani16142161

**Published:** 2026-07-12

**Authors:** Yuhua Lv, Yuexia Lin, Yue Liu, Shuzhen Yang, Wanhe Du, Rongrong Liao

**Affiliations:** 1 Institute of Animal Husbandry and Veterinary Science, Shanghai Academy of Agricultural Sciences, Shanghai 201106, China; 2 School of Intelligent Manufacturing and Control Engineering, Shanghai Polytechnic University, Shanghai 201209, China

**Keywords:** Chongming white goat, estrus, reproductive hormones, vision-based monitoring, reproduction

## Abstract

Vision-based estrus monitoring for goats has matured rapidly, yet endocrine preliminary feasibility evaluation in indigenous breeds is largely missing. Here we conducted a preliminary feasibility evaluation of whether behavioral estrus events captured by a machine vision pipeline align with reproductive endocrine dynamics in Chongming white goats. Ten multiparous does were monitored continuously for 21 days under standard housing conditions; daily jugular samples yielded 206 paired serum measurements of estradiol (E2), FSH, LH, and progesterone (P4). Behavioral events were extracted from infrared video by a YOLOv8-seg + MMPose pipeline and cross-checked against teaser-buck confirmation. Hormone profiles differed markedly between estrus and non-estrus windows (all *p* < 0.001): estradiol rose from 14.81 ± 4.17 to 21.79 ± 2.10 ng/L, and progesterone fell from 2386.70 ± 466.93 to 1719.68 ± 157.52 pmol/L. Estrus status correlated moderately with E2 (r = 0.459), FSH (r = 0.435), and LH (r = 0.446), and inversely with P4 (r = −0.406); the E2/P4 ratio produced the most pronounced per-event peak and is proposed as a preliminary sensitive auxiliary indicator. Vision-based detection reached 86.96% accuracy. The data indicate that camera-derived behavioral markers track real physiological state changes in this breed, supporting the deployment of vision-based estrus screening as a first-pass tool, with hormone assays reserved for confirmation in commercial Chongming white goat herds.

## Simple Summary

Effective identification of estrus onset remains a practical bottleneck in Chongming white goat breeding, where missed detections translate directly into extended kidding intervals and economic loss. Manual observation and single-parameter sensors cannot accurately capture complex estrus behaviors. To address this, we deployed an infrared-camera-based machine vision pipeline built on YOLOv8-seg and MMPose, monitoring ten multiparous does over 21 days. Serum concentrations of estradiol, FSH, LH, and progesterone were quantified daily by ELISA and aligned in time with the video-derived behavioral events. Four key findings emerged: hormone profiles differed sharply between estrus and non-estrus windows (all *p* < 0.001); behavioral estrus correlated moderately with E2 (r = 0.459), FSH (r = 0.435), and LH (r = 0.446), and inversely with progesterone (r = −0.406); the E2/P4 ratio showed a pronounced peak around each estrus event; and the vision system reached 86.96% accuracy against teaser-buck-confirmed ground truth. In this single-cycle, small-cohort setting, this accuracy should be interpreted as a preliminary benchmark rather than a generalizable performance estimate. The results provide a quantitative basis for deploying camera-based estrus screening on Chongming white goat farms.

## 1. Introduction

Accurate estrus detection is a core component of successful reproductive management in goat production systems, directly affecting conception rates, kidding intervals, and overall herd productivity. The ability to identify estrus and target optimal breeding windows is a fundamental determinant of reproductive success; delays or missed detections can cause significant economic losses by extending kidding intervals and reducing annual reproductive output [1,2]. Estrus behavior indicators in does include a range of characteristic changes: increased activity, tail wagging (tail raising), arched back posture, reduced feed intake, increased vocalization, and actively approaching bucks [3,4]. Estrus duration typically ranges from 24 to 48 h, depending on follicular development dynamics and individual variation [5,6]. These behaviors are mediated by dynamic changes in gonadotropin and steroid hormone concentrations via the hypothalamic-pituitary-ovarian (HPO) axis [7]. In early estrus, follicle-stimulating hormone (FSH) promotes follicular development and selection, laying the foundation for subsequent estrogen secretion. As follicles mature, estradiol (E2) levels secreted by developing follicles rise, triggering typical estrus behaviors [8,9]. The preovulatory luteinizing hormone (LH) surge induces final follicular maturation and ovulation, with FSH also participating in regulating LH receptor expression during this process [10,11]. Meanwhile, progesterone (P4) levels are low during estrus due to luteal regression, then gradually rise during the luteal phase [12]. Studies indicate that the synergistic action of E2 and LH is not only key to ovulation but also a potential biomarker for quantifying estrus intensity [13].

In traditional farming systems, estrus detection has largely depended on manual observation or the use of teaser bucks. The reliability of these methods is highly contingent on handler experience, observation frequency, and environmental conditions such as temperature, lighting, and stocking density, leading to high labor demands, subjectivity, and suboptimal accuracy. Some larger-scale goat farms have begun exploring automated solutions. Broadly, automated estrus detection in livestock has evolved along two complementary technological trajectories. The first trajectory relies on wearable sensors—including accelerometers, pedometers, rumination boluses, and ear-tag thermometers—which provide continuous, individual-level physiological or behavioral proxies and have been successfully applied to estrus detection in dairy cattle, sheep, and goats [14,15,16,17,18,19]. The second trajectory employs machine vision, using fixed or mobile cameras coupled with deep-learning-based pose estimation and behavior recognition, which enables non-contact, multi-animal, 24 h behavioral monitoring without the need for device attachment [20,21,22,23]. These two approaches differ in their operational profiles rather than being mutually exclusive: wearables typically offer higher per-animal temporal resolution but require device handling, battery management, and may be susceptible to loss or damage; vision systems avoid animal handling and can capture complex, multi-animal social interactions, but they depend on fixed camera infrastructure, are sensitive to lighting and occlusion, and demand non-trivial computational resources for real-time inference. Deploying computer vision systems in precision livestock farming presents considerable challenges. Effective implementation requires substantial infrastructure, including optimal camera placement to minimize animal occlusion, consistent lighting conditions (often necessitating infrared supplementation for nighttime monitoring), and significant computational power for real-time video processing. A balanced approach that acknowledges these environmental and hardware dependencies is therefore crucial for translating visual algorithms into practical farm management.

The present study adopts the vision-based approach, motivated by two key considerations: the need to detect complex social behaviors—such as mounting, standing-to-be-mounted, and sustained tail elevation—which single-parameter sensors cannot easily disambiguate, and the goal of scaling behavioral monitoring to group-housed commercial herds. However, research on vision-based estrus identification in goats remains scarce, particularly regarding its validation against reproductive endocrine indicators. To address this gap, we focused on the Chongming white goat, one of the most important native breeds in Shanghai, China, known for its adaptability, high reproductive efficiency, and superior meat quality [24,25]. In recent years, the rapid expansion and intensification of goat farming have imposed greater demands on daily management, disease control, and welfare monitoring. Concurrently, Shanghai is advancing the digital and intelligent transformation of urban agriculture, with the goal of establishing a model for facility-based animal husbandry. Intelligent estrus monitoring for does fits naturally into this trajectory. There is thus an urgent need to develop non-invasive, machine vision-based systems that can replace labor-intensive manual observation. Such systems should not only automatically identify estrus behaviors and assess reproductive status, but also coordinate with barn environmental controls (e.g., light, temperature, and humidity) to promote estrus expression and overall health. Ultimately, this integrated approach is expected to improve estrus detection rates and breeding success [22,26]. Accordingly, we set two primary goals for this study. The first is to develop and deploy a vision-based estrus detection pipeline that is specifically adapted to the behavioral patterns of Chongming white goats. The second is to verify the system’s biological accuracy by continuously aligning its behavioral classifications with precise temporal measurements of reproductive hormones (E2, FSH, LH, P4). Through this integration of machine vision and endocrine reference data, we aim to provide preliminary support for the potential use of automated estrus screening in commercial goat production.

## 2. Materials and Methods

### 2.1. Ethics Approval and Consent to Participate

The research protocol for this study was approved by the Ethics and Animal Welfare Committee of Shanghai Academy of Agricultural Sciences (Approval No. SAASPZ0526174). All blood collection procedures were performed by experienced personnel using appropriate restraint methods to minimize animal stress and discomfort.

### 2.2. Experimental Animals and Management

For this study, ten multiparous, healthy, non-pregnant Chongming white goat does, aged 2–4 years and with body weights of 40–45 kg, were selected. All does were housed at the Chongming white goat experimental station of Shanghai Academy of Agricultural Sciences on Chongming Island, Shanghai. Inclusion criteria were at least two kidding records to demonstrate fertility, confirmed normal estrous cycles, and absence of clinical reproductive disorders or systemic diseases at enrollment. During the experiment, every two does were kept together in a pen (2.5 m × 1.8 m). The barn temperature was maintained at 10–18 °C, and the animals had free access to water and a standard diet (see Supplementary Table S1 for the diet formulation). This study was conducted from 7 December to 27 December 2025. The study paddocks were located approximately 80 m from the buck barn, and direct contact with bucks was prevented throughout the 21-day trial except for the two scheduled teaser buck confirmation windows (08:30 and 16:30), to avoid pheromone-induced early estrus that could confound behavioral interpretation. The experiment involved 21 consecutive days of machine vision observation and sampling; this duration was chosen because the estrous cycle of goats is typically 17–21 days, with estrus lasting 24–48 h, ensuring at least one full estrous cycle was covered. Health status was monitored daily throughout the experimental period; any animal showing signs of stress or illness was immediately withdrawn from the study.

### 2.3. Blood Collection and Hormone Assay

Each day between 07:00 and 08:00, approximately 2 mL of blood was drawn from the jugular vein of each doe using vacuum tubes. To minimize circadian interference with hormone baselines, sampling was strictly confined to this 1 h morning window throughout the 21-day trial. Tubes were immediately chilled on wet ice, delivered to the on-station laboratory within 30 min of collection, and centrifuged at 3000× *g* and 4 °C for 15 min. The serum fraction was aliquoted into pre-labeled 1.5 mL cryovials and stored at −80 °C until batch assay. Concentrations of E2, FSH, LH, and P4 were quantified with commercial goat-validated ELISA kits (Nanjing Jiancheng Bioengineering Institute, Nanjing, China). To reduce inter-assay drift, all samples from a single animal were measured on the same plate whenever possible; otherwise, samples were balanced across plates by estrus status. Each kit included its own standard curve, and the manufacturer-reported intra- and inter-assay coefficients of variation were accepted as published. All assays were performed by a single trained technician to limit operator-level variability.

### 2.4. Machine Vision-Based Estrus Detection

**Figure 1:**
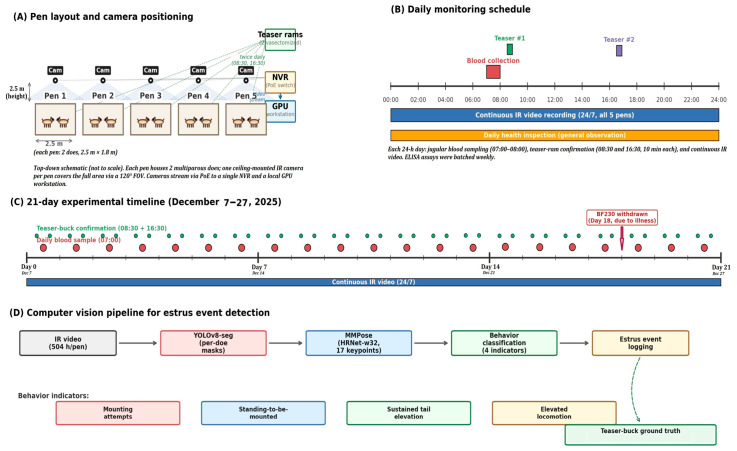
Schematic of the experimental design. (**A**) Pen layout and camera positioning. (**B**) Daily monitoring schedule. Each 24 h day included jugular blood sampling, twice-daily teaser-buck confirmation (08:30 and 16:30, 10 min each), and continuous IR video recording. (**C**) 21-day experimental timeline. (**D**) Computer vision pipeline for estrus detection.

The experimental design and model training workflow of the machine vision-based estrus detection system are presented in the following sections and are shown schematically in Figure 1.

Image acquisition and preprocessing. Each pen was fitted with one infrared network camera (Hikvision, Hangzhou, China) mounted at 2.5 m above floor level with a 120° field of view, chosen so that the full pen area remained within frame under both daylight and zero-illumination nighttime conditions. Continuous recording ran for the entire 21-day trial, producing approximately 504 h of video per pen. From the massive surveillance footage, high-definition video frames were extracted using a stratified sampling strategy to cover varying lighting conditions and distinct behavioral postures—including mounting, standing, and tail elevation—yielding a custom raw dataset comprising 1200 images of Chongming white goats.

Dataset annotation. To meet the requirements of a “top-down” vision pipeline, the dataset underwent fine-grained two-level annotation. First, a polygon tool was used to annotate pixel-wise instance masks for each goat in every image, enabling the subsequent YOLOv8-seg model to accurately segment individual goat silhouettes and eliminate background noise from pen enclosures and overlapping animals. Second, on the segmented goat bodies, 2D spatial coordinates were labeled for key anatomical landmarks, including the head, spine, limbs, and tail base, providing a spatial topology for the MMPose model. The fully annotated 1200-image dataset was then strictly divided into training, validation, and test sets at an 8:1:1 ratio to prevent overfitting.

Model training and optimization. All training and optimization were conducted on a high-performance workstation equipped with an NVIDIA RTX 3090 GPU (NVIDIA, CA, USA), running Python 3.8, PyTorch 2.0.0, and the OpenMMLab 2.0 ecosystem. The training proceeded in two stages. In the first stage, YOLOv8-seg was trained on the training set for instance segmentation, with a focus on optimizing the feature pyramid network and mask generation branches. This enabled the model to output high-signal-to-noise ratio target regions for individual goats, replacing traditional rectangular bounding boxes and effectively resolving visual challenges caused by group housing and overlapping animals. In the second stage, the cropped high-quality single-goat images were fed directly into the MMPose framework for 2D coordinate regression training, allowing the model to accurately localize the spatial positions of limbs and tail base based on pixel features. Evaluation metrics—including Average Recall (AR), Average Precision (AP), and AP at IoU = 0.5 (AP_50_)—were computed on the validation and test sets, and hyperparameters such as learning rate and batch size were iteratively adjusted until the accuracy of both models met the deployment requirements for practical farm settings. The final performance on the validation and test sets was as follows: YOLOv8-seg achieved AP 0.601, AP_50_ 0.631, and AR 0.766; MMPose attained AP 0.836, AP_50_ 0.931, and AR 0.869.

Deployment, behavioral rule engine, and estrus determination. Once the models converged and began outputting high-precision coordinate data, the system entered the deployment phase, where a spatiotemporal behavioral rule engine was activated. In practice, the system is fully activated during a predefined “post-weaning observation window” based on conventional husbandry experience. The detection pipeline operated as follows: individual does were segmented frame-by-frame with a YOLOv8-seg model [27,28], which yields pixel-level masks rather than bounding boxes and therefore tolerates partial occlusions from pen mates and feeding barriers typical of goat housing. The cropped high-signal-to-noise patches were then passed to an MMPose-based [29,30] 2D keypoint regressor, which reconstructed the body topology (head, spine, limbs, tail base) of each animal; estrus-relevant behaviors—mounting attempts, standing-to-be-mounted, sustained tail elevation, and elevated locomotion—were derived from the temporal evolution of this topology across consecutive frames. Behavioral actions were quantified through preset geometric and spatiotemporal rules. For example, when the forelimb keypoints of one doe spatially overlapped with the hindquarters of another doe across consecutive video frames for a duration exceeding a set threshold, the algorithm provisionally flagged it as a “mounting attempt.” An estrus event was defined as a cluster of at least two co-occurring behavioral indicators within any rolling 24 h window, provided that standing reflex or accepting-mounting was flagged with confidence above a preset threshold; the timestamp and duration of each such event were logged automatically. The first day on which the algorithm-flagged event coincided with teaser buck confirmation (see below) was defined as estrus day 0 for that animal. This workflow completes the full closed-loop pipeline—from raw environmental image acquisition, front-end feature denoising, and precise coordinate regression, to final physiological state prediction.

Validation with teaser bucks. To verify the accuracy of the machine vision system for estrus determination, teaser (vasectomized) bucks were introduced twice daily (08:30 and 16:30) for auxiliary confirmation. Within 10 min of teaser buck contact with the doe herd, two experienced observers recorded whether does exhibited standing reflex and typical estrus behaviors. If a doe stood motionless when mounted by the teaser buck within the estrus window marked by the vision system, and simultaneously showed at least two other behaviors (such as tail wagging, vocalization, vulvar swelling, etc.), it was manually confirmed as true estrus; otherwise, it was considered suspected or falsely determined pseudo-estrus, used for feedback to optimize the vision recognition model. The day when a doe’s first estrus was manually confirmed and matched the vision recognition result was defined as estrus day 0.

### 2.5. Statistical Analysis

After initial organization and verification in Microsoft Excel spreadsheets, independent samples *t*-tests were used to compare hormone concentrations between estrus and non-estrus periods, with statistical significance set at *p* < 0.05. Pearson correlation coefficients were calculated to assess associations among the four hormones (E2, FSH, LH, and P4) serum concentrations. Correlation analysis was based on all available paired observations during the experimental period. All statistical analyses were performed using IBM SPSS Statistics software (version 19.0, IBM Corp., Armonk, NY, USA). Results are expressed as mean ± standard deviation unless otherwise stated.

## 3. Results

### 3.1. Vision Pipeline Performance Evaluation

**Figure 2:**
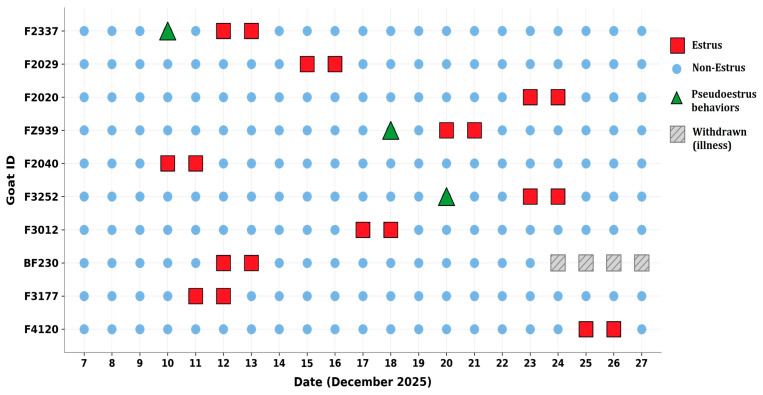
Distribution of estrus, pseudoestrus, and non-estrus days across 10 does over the 21-day experimental period. Rows represent individual goat IDs (*n* = 10), and columns represent observation days (7–27 December 2025). Red cells indicate days when estrus behavior was observed; blue cells indicate non-estrus periods; green triangles indicate pseudoestrus behaviors; diagonal stripes indicate withdrawal from the study due to illness.

Prior to correlating behavioral metrics with hormonal changes, the efficacy of the customized computer vision system was evaluated against the teaser-buck ground truth. During the 21-day observation period, all 10 does exhibited a single estrus period (lasting 1–2 days), within which 23 discrete behavioral estrus episodes were recorded (mean 2.3 episodes per animal; range 2–3). The multiple episodes within individual does reflect repeated behavioral peaks within a single estrus period. Three does each exhibited one pseudo-estrus episode, which generally appeared 1–3 days before true estrus (Figure 2). Over the 21-day trial, the integrated YOLOv8-seg and MMPose pipeline achieved an overall estrus detection accuracy of 86.96%. The algorithm demonstrated a sensitivity of 87.0% and a high specificity of 94.2% in distinguishing true standing reflexes and sustained mounting from incidental pen activities. The most frequently observed behaviors during estrus included: tail wagging (tail raising, 100% of estrus events), increased vocalization (85%), mounting or attempting to mount other does (70%), restlessness (75%), vulvar swelling with discharge (60%), and reduced appetite (65%). Standing estrus when exposed to teaser bucks was observed in 90% of confirmed estrus events. Two does exhibited estrus behavioral manifestations but did not show clear standing estrus. One doe was monitored normally through estrus but withdrew from monitoring and blood collection in the last 4 days of the experiment due to illness.

The accuracy of the proposed vision-based method was benchmarked against that of conventional approaches. The vision system attained 86.96% accuracy, whereas the teaser-buck method achieved 85%, and manual observation, serving as the ground truth, provided 100% accuracy.

### 3.2. Hormone Levels in Estrus vs. Non-Estrus States

**Figure 3:**
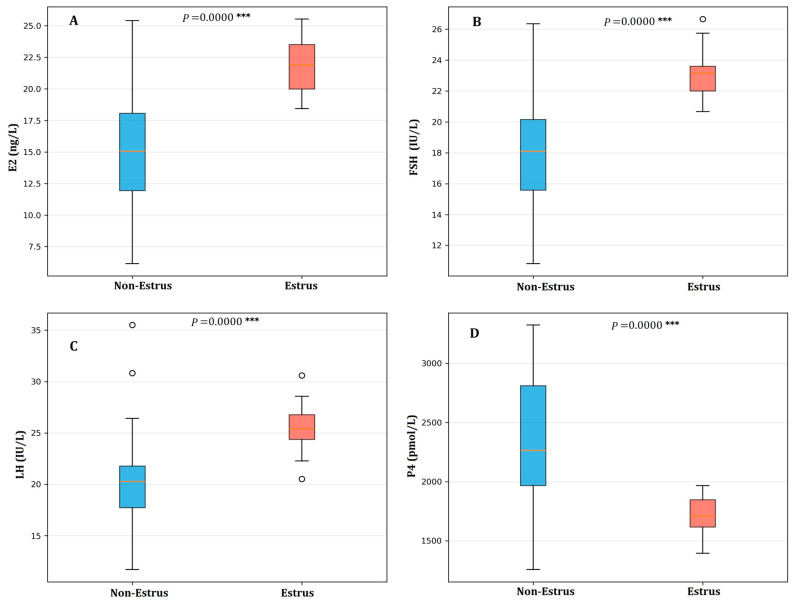
Box plot comparison of hormone concentrations between estrus (red) and non-estrus (blue) behavioral states. Panels show: (**A**) Estradiol, (**B**) FSH, (**C**) LH, and (**D**) Progesterone. *** denotes *p* < 0.001.

The manufacturer-reported intra-assay coefficients of variation were ≤9.1% for E2, ≤9.8% for FSH, ≤9.6% for LH, and ≤9.2% for P4; inter-assay CVs were ≤10.5% for E2, ≤10.8% for FSH, ≤10.5% for LH, and ≤11.1% for P4. Our observed intra-assay CVs were 6.9% (E2), 7.2% (FSH), 7.5% (LH), and 6.6% (P4); inter-assay CVs were 9.5% (E2), 9.8% (FSH), 9.6% (LH), and 8.9% (P4), all consistent with manufacturer specifications. Significant differences in hormone profiles were observed between estrus and non-estrus behavioral periods (Table 1). Estradiol concentrations were significantly elevated during estrus (21.79 ± 2.10 ng/L) compared to non-estrus (14.81 ± 4.17 ng/L), an increase of 47% (t = 7.378, *p* < 0.001). FSH showed a similar pattern, with estrus concentration at 23.02 ± 1.50 IU/L versus 17.73 ± 3.38 IU/L during non-estrus (t = 6.900, *p* < 0.001). LH increased from 19.71 ± 3.50 IU/L during non-estrus to 25.43 ± 2.33 IU/L during estrus (t = 7.126, *p* < 0.001). In contrast, progesterone concentrations significantly decreased during estrus to 1719.68 ± 157.52 pmol/L compared to 2386.70 ± 466.93 pmol/L during non-estrus (t = −6.338, *p* < 0.001) (Figure 3).

### 3.3. Inter-Hormone Correlations

**Figure 4:**
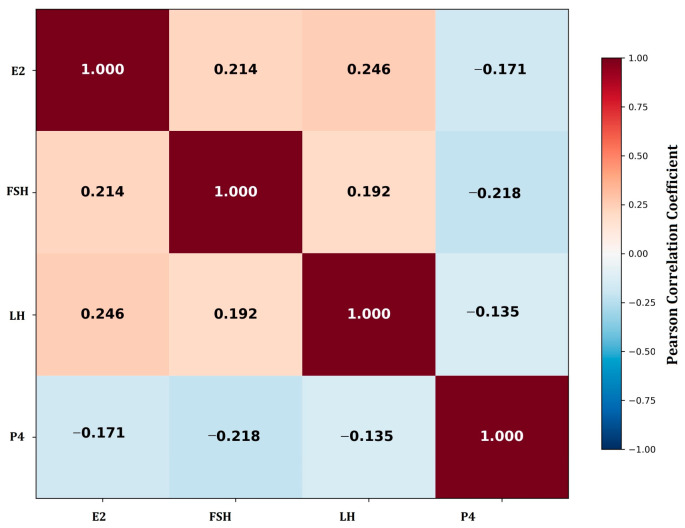
Inter-hormone Pearson correlation matrix heatmap. Color intensity indicates correlation strength, with red representing positive correlations and blue representing negative correlations. Values within cells show exact correlation coefficients.

Correlation analysis among the four hormones showed weak positive correlations between estradiol and FSH (r = 0.214) and LH (r = 0.246) (Figure 4), consistent with their coordinated secretion during the follicular phase. Progesterone showed weak negative correlations with the other three hormones, consistent with corpus luteum dominance during periods when follicular hormones decline.

### 3.4. Correlation Between Estrus Status and Hormones

Correlation analysis revealed significant relationships between estrus status and all hormone variables (Table 2). Moderate positive correlations were observed with estradiol (r = 0.459, *p* < 0.001), FSH (r = 0.435, *p* < 0.001), and LH (r = 0.446, *p* < 0.001). Progesterone showed a moderate negative correlation with estrus status (r = −0.406, *p* < 0.001).

### 3.5. E2/P4 Ratio as an Estrus Indicator

**Figure 5:**
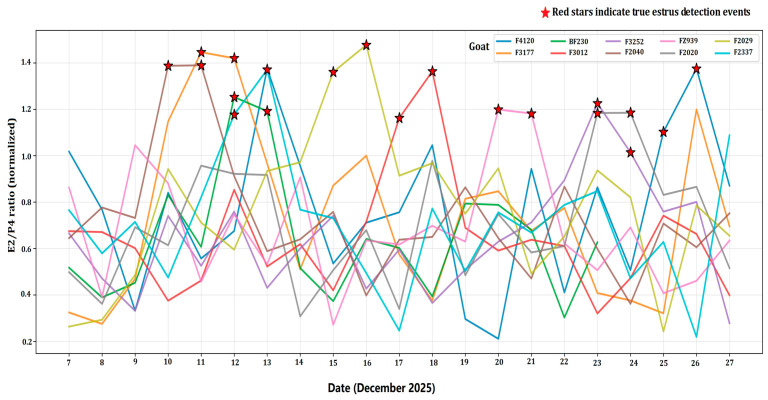
E2/P4 ratio patterns for each goat throughout the observation period. Red stars indicate true estrus detection events. Ratio values are normalized (P4 divided by 100) to enable comparable scaling with E2.

The estradiol-to-progesterone ratio showed significant elevation during estrus events. Examination of E2/P4 ratio trajectories revealed typical peak patterns before, during, and after estrus detection (Figure 5). Individual variation existed in ratio magnitude and timing, with some goats showing sharp instantaneous peaks and others showing more sustained pulsatile elevations.

## 4. Discussion

### 4.1. Performance Evaluation of Vision-Based Estrus Detection

The video acquisition module in this study used infrared network cameras for round-the-clock continuous monitoring of does. The segmentation network employed the YOLOv8-seg model [27,28], which, compared to traditional object detection that only outputs rectangular bounding boxes, can precisely extract pixel-level contours of individual goats, effectively removing background noise such as pen fencing and other overlapping goats. After obtaining target masks, the system adopted a top-down strategy, inputting cropped high signal-to-noise ratio target regions into the pose keypoint extraction network. This network was constructed based on the MMPose pose estimation framework [29,30], and performed two-dimensional coordinate regression on core keypoints such as limbs and torso of does, constructing a spatial topology map representing their posture, and ultimately precisely identifying and extracting typical estrus behavioral features such as mounting based on temporal evolution patterns of keypoint topology structures in consecutive video frames.

The strength of the association between estrus behavioral indicators and hormone measurements is noteworthy [31]. Although moderate correlation coefficients (r ≈ 0.40–0.46) may suggest considerable unexplained variation, this magnitude is consistent with the multifactorial complexity of estrus behavior [7,10]. Thresholds for individual behavioral expression, environmental influences, and measurement timing relative to LH peaks all contribute to behavioral-hormone relationship variability [10]. Therefore, this study validated a vision-based estrus detection method applicable to Chongming white goats by analyzing the correlation between intelligent estrus identification and serum reproductive hormone profiles. Results showed that visual behavioral indicators during the estrous cycle showed moderate alignment with endocrine changes, suggesting that vision-based detection systems may serve as a preliminary screening tool for estrus identification in Chongming white goats. The moderate correlations (r = 0.406–0.459) between estrus status and the four hormones suggest that visual observation may reflect physiological state changes, although these correlations alone do not confirm a genuine transition or fully exclude sporadic behavioral variations. Notably, the correlations observed in this study exceeded those typically reported for indirect estrus indicators in other livestock by Dolecheck et al. [31] and Lodkaew et al. [32]. The machine vision-based estrus detection method in this study achieved promising preliminary accuracy, somewhat improved compared to traditional manual observation methods [15]. These results suggest potential for integrating vision-based estrus detection systems into Chongming white goat farming and commercial reproductive management programs, though further validation with larger cohorts is warranted.

### 4.2. Hormonal Profiles and Estrus Cycle Dynamics

Endocrine regulation of the goat estrous cycle involves complex interactions among the hypothalamus, pituitary, and ovaries [7]. Estradiol, produced by developing follicles, plays a central role in stimulating behavioral estrus expression by acting on the central nervous system. FSH and LH coordinate follicular development and ovulation by acting on ovarian receptors [33]. Progesterone, primarily secreted by the corpus luteum after ovulation, maintains uterine conditions suitable for pregnancy establishment and exerts negative feedback on gonadotropin secretion during the luteal phase [34]. The hormone variation patterns observed in this study are consistent with established understanding of small ruminant estrous cycle physiology. The significant elevation of estradiol during estrus (approximately 47% higher than non-estrus) reflects preovulatory follicular activity and drives receptive behavioral expression. Estradiol acts on the central nervous system, regulating neurotransmitter systems that control sexual behavior, explaining the tight temporal correspondence between hormone elevation and behavioral expression [13].

The increases in FSH and LH during estrus similarly reflect pituitary responsiveness to decreased progesterone negative feedback and increased estradiol positive feedback [35]. The approximately 30% increase in LH concentration during estrus is consistent with recorded preovulatory peak magnitudes in this species [8]. The approximately 28% increase in FSH complements follicular recruitment processes for transition to the next cycle. The dramatic decline in progesterone during estrus—from over 2300 pmol/L to below 1800 pmol/L—confirms corpus luteum regression preceding behavioral estrus. This progesterone withdrawal removes behavioral inhibition and allows estrus characteristic expression [8]. The negative correlation between P4 and estrus status (r = −0.406) confirms the central role of corpus luteum regression in estrus timing.

### 4.3. Inter-Hormone Relationships

Weak to moderate positive correlations (r = 0.19–0.25) among E2, FSH, and LH reflect their coordinated secretion during the follicular phase while maintaining consistency with independent regulatory patterns [33,34]. The observed correlation magnitudes fall within reported ranges for this species, where pulsatile secretion patterns and diurnal variation introduce measurement variability. Negative correlations between progesterone and the other three hormones confirm the alternating dominance of follicular and luteal phase endocrine environments.

From these data, the E2/P4 ratio emerges as a particularly informative indicator. The inverse hormone dynamics—estradiol elevation combined with progesterone decline—amplify the ratio difference between estrus and non-estrus states, with effects exceeding those of either hormone alone. Practical applications might consider using E2/P4 thresholds for automated estrus alerts, with ratio elevation potentially serving as a candidate confirmation of vision-based detection.

### 4.4. Practical Implications of Vision-Based Estrus Detection

Crucially, while robust pose recognition and multi-modal feature extraction served as essential preliminary work, the primary goal of this study was automated estrus detection. The present study advances beyond existing binary detection systems by demonstrating a direct, quantitative dose–response relationship between algorithmic outputs and hormonal blood profiles. The significant correlations between keypoint-derived behavioral frequencies (e.g., mounting, tail elevation) and E2/P4 dynamics confirm that the neural network’s detection intensity inherently scales with the physiological intensity of the estrus event. This suggests that the computer vision pipeline effectively functions as a non-invasive optical biosensor. Consequently, the depth of behavioral data extracted by the system offers a resolution capable of predicting peak follicular activity, providing farm managers with a graded physiological assessment rather than a simple on/off alert.

Beyond its contribution to reproductive physiology, the vision pipeline also demonstrates practical advantages over conventional methods. Compared with manual teaser-ram observation, it achieved comparable sensitivity (86.96% vs. 85%) while reducing labor input from approximately 60 min/day to a negligible post-deployment cost. In the context of reproductive biotechnology, the pipeline can be integrated with timed artificial insemination (AI) protocols by detecting does entering standing estrus in real time. It could also support embryo transfer donor–recipient synchronization by aligning the peak E2/P4 windows across animals. These findings support the integration of vision-based estrus detection into routine management programs for Chongming white goats, as the demonstrated correspondence between behavioral indicators and hormonal status validates its feasibility. Key advantages include continuous monitoring, elimination of labor for manual checks, and consistent sensitivity for simultaneous multi-animal detection.

Successful implementation, however, requires attention to several technical considerations. Camera positioning must ensure complete coverage of areas where estrus behaviors manifest, particularly mounting and standing-to-be-mounted zones. Lighting conditions, including infrared illumination for nighttime observation, directly impact detection reliability. Algorithm development for automated behavioral analysis should incorporate the characteristic indicators documented in this study, including arched back posture, standing to accept mounting, increased activity, and tail movement. Deep learning methods—particularly convolutional neural networks and pose estimation frameworks such as YOLOv8 and MMPose—have shown great potential in livestock behavioral analysis [16,17]. Combining these technologies with traditional physiological indicators is expected to further improve detection accuracy and reduce false alarm rates [18,19].

### 4.5. Limitations and Future Directions

Although the vision-based estrus detection method in this study achieved a promising detection rate, several limitations remain. First, the relatively small sample size (n = 10) limits generalizability, although the intensive longitudinal design partially compensates through multiple within-animal observations. Second, the observation period covered only one estrous cycle without accounting for seasonal variation, which may not represent the full range of hormonal patterns across the breeding season and cannot address potential seasonal changes in hormone-behavior relationships [36,37]; future research should monitor consistency across multiple estrous cycles and seasons. Third, the daily hormone sampling frequency may mask important temporal dynamics within estrous cycles, and is unable to capture acute fluctuations such as LH pulses that last only a few hours, and sampling was conducted only in the morning, potentially missing critical changes at other times of day. Fourth, we recognize that housing arrangement with only two does per paddock differs from standard farm practices. Fifth, environmental dependency of the vision-based estrus detection: the 86.96% sensitivity reported here was obtained under the controlled conditions of the present study (single 2.5 m × 1.8 m pen per camera, paired housing, IR-supplemented nighttime illumination, no direct sunlight exposure). Performance under commercial farming conditions may be reduced by several environmental factors: (i) lighting variability—direct sunlight glare and shadow flicker can degrade segmentation masks; (ii) occlusion—group sizes of 8–20 does per pen (typical in commercial Chongming white goat herds) increase the frequency of multi-animal mounting clusters, raising the per-frame occlusion rate; and (iii) camera infrastructure—dust, lens contamination, and condensation can degrade image quality over multi-month deployment.

Future research should advance in the following directions: (1) Expand sample size and conduct longitudinal studies across breeding seasons to track hormone-behavior consistency in individuals across multiple estrous cycles and years; (2) Employ higher frequency sampling schemes (such as every 4–6 h) or continuous hormone monitoring technologies to precisely characterize hormone peak timing and its relationship with behavioral onset; (3) Integrate hormone and behavioral data streams, leveraging machine learning algorithms to improve detection accuracy; (4) Incorporate covariates such as parity, body condition, and nutrition to enhance understanding of individual differences in reproductive physiology; (5) Refine and scale up the recognition method to enable estrus detection in larger groups under practical production settings.

## 5. Conclusions

In this preliminary, single-cycle study involving 10 does over 21 days, a YOLOv8-seg + MMPose vision pipeline detected estrus events with 86.96% agreement against teaser-buck ground truth. These events appeared broadly consistent with the predicted endocrine shifts: a 47% rise in estradiol, a 28% decline in progesterone, and coordinated increases in FSH and LH, with the E2/P4 ratio emerging as the most temporally localized indicator. Moderate correlation coefficients (r ≈ 0.41–0.46) between vision-derived estrus status and each of the four hormones suggest that camera-based behavioral markers may reflect underlying physiological changes, although they do not by themselves confirm a definitive state transition.

Two preliminary considerations emerge from these findings. First, the pipeline may serve as a labor-saving first-pass screening tool in commercial Chongming white goat herds, with serum hormone assays reserved for ambiguous cases. Second, the E2/P4 ratio appears to be a candidate feature for embedding directly into detection algorithms to potentially suppress false positives during the luteal-phase transition. However, both directions require evaluation across multiple estrous cycles and breeding seasons before any herd-level deployment can be recommended.

## Figures and Tables

**Table 1:** Comparison of serum hormone concentrations between estrus and non-estrus behavioral states.

Hormone	Estrus	Non-Estrus	*p*-Value
E2 (ng/L)	21.79 ± 2.10	14.81 ± 4.17	<0.001
FSH (IU/L)	23.02 ± 1.50	17.73 ± 3.38	<0.001
LH (IU/L)	25.43 ± 2.33	19.71 ± 3.50	<0.001
P4 (pmol/L)	1719.68 ± 157.52	2386.70 ± 466.93	<0.001

**Table 2:** Pearson correlation coefficients between estrus status and serum hormone concentrations.

Variable Pair	Correlation (r)	*p*-Value	Interpretation
Estrus vs. E2	0.459	<0.001	Moderate positive
Estrus vs. FSH	0.435	<0.001	Moderate positive
Estrus vs. LH	0.446	<0.001	Moderate positive
Estrus vs. P4	−0.406	<0.001	Moderate negative

## Data Availability

The original contributions presented in the study are included in the article/Supplementary Material; further inquiries can be directed to the corresponding authors.

## Supplementary Materials

The following supporting information can be downloaded at: https://www.mdpi.com/article/10.3390/ani16142161/s1, Table S1. Feed composition and nutrient levels of Chongming white goat diets (air-dry basis).

## Abbreviations

The following abbreviations are used in this manuscript:

E2	Estradiol
FSH	Follicle-stimulating hormone
LH	Luteinizing hormone
P4	Progesterone

## References

[1] Endo N., Rahayu L.P., Arakawa T., Tanaka T. (2016). Video tracking analysis of behavioral patterns during estrus in goats. J. Reprod. Dev..

[2] Reith S., Hoy S. (2018). Review: Behavioral signs of estrus and the potential of fully automated systems for detection of estrus in dairy cattle. Animal.

[3] González-Tavizón A., Meza-Herrera C.A., Arellano-Rodríguez G., Mellado M., Contreras-Villarreal V., Ángel-García O., Arévalo J.R., Véliz-Deras F.G. (2022). Effect of Dorper rams’ social-sexual hierarchy on their sexual behavior and capacity to induce estrus in ewes. Agriculture.

[4] Habeeb H.M.H., Anne Kutzler M. (2021). Estrus synchronization in the sheep and goat. Vet. Clin. N. Am. Food Anim. Pract..

[5] Romano J.E., Keisler D.H., Amstalden M. (2018). Effect of copulation on estrus duration, LH response, and ovulation in Boer goats. Theriogenology.

[6] Romano J.E., Alkar A., Amstalden M. (2016). Effect of copulation on estrus duration and ovulation time in goats. Theriogenology.

[7] Gaafar K.M., Gabr M.K., Teleb D.F. (2005). The hormonal profile during the estrous cycle and gestation in Damascus goats. Small Rumin. Res..

[8] Khanum S., Hussain M., Kausar R. (2008). Progesterone and estradiol profiles during estrous cycle and gestation in dwarf goats (*Capra hircus*). Pak. Vet. J..

[9] Sohail T., Farhab M., Zhang L., Kang Y., Sun X., Ji D., Li Y. (2023). Ovarian Dynamics and Changes in Estradiol-17β and Progesterone Relationship with Standing Estrus, Preovulatory Follicles, and Ovulation Using Single Prostaglandin F2α Injection in Barbari Goats. Vet. Sci..

[10] Abecia J., Forcada F., González-Bulnes A. (2012). Hormonal control of reproduction in small ruminants. Anim. Reprod. Sci..

[11] Zhu Y., Ye J., Qin P., Yan X., Gong X., Li X., Liu Y., Li Y., Yu T., Zhang Y. (2023). Analysis of serum reproductive hormones and ovarian genes in pubertal female goats. J. Ovarian Res..

[12] Menchaca A., Rubianes E. (2002). Relation between progesterone concentrations during the early luteal phase and follicular dynamics in goats. Theriogenology.

[13] Martin O., Friggens N.C., Dupont J., Salvetti P., Freret S., Rame C., Elis S., Gatien J., Disenhaus C., Blanc F. (2013). Data-derived reference profiles with corepresentation of progesterone, estradiol, LH, and FSH dynamics during the bovine estrous cycle. Theriogenology.

[14] Talukder S., Thomson P.C., Kerrisk K.L., Clark C.E., Celi P. (2015). Evaluation of infrared thermography body temperature and collar-mounted accelerometer and acoustic technology for predicting time of ovulation of cows in a pasture-based system. Theriogenology.

[15] Ding L., Zhang C., Yue Y., Yao C., Li Z., Hu Y., Yang B., Ma W., Yu L., Gao R. (2025). Wearable Sensors-Based Intelligent Sensing and Application of Animal Behaviors: A Comprehensive Review. Sensors.

[16] Arakawa T. (2020). Possibility of Autonomous Estimation of Shiba Goat’s Estrus and Non-Estrus Behavior by Machine Learning Methods. Animals.

[17] Sharifuzzaman M., Mun H.S., Ampode K.M.B., Lagua E.B., Park H.R., Kim Y.H., Hasan M.K., Yang C.J. (2024). Technological Tools and Artificial Intelligence in Estrus Detection of Sows-A Comprehensive Review. Animals.

[18] Wang J., Bell M., Liu X., Liu G. (2020). Machine-Learning Techniques Can Enhance Dairy Cow Estrus Detection Using Location and Acceleration Data. Animals.

[19] Marques T.C., Marques L.R., Fernandes P.B., de Lima F.S., do Prado Paim T., Leão K.M. (2024). Machine Learning to Predict Pregnancy in Dairy Cows: An Approach Integrating Automated Activity Monitoring and On-Farm Data. Animals.

[20] Liu H., Li H., Cao Y., Cao R., Hu G., Liu Z. (2026). Edge-AI Enabled Acoustic Monitoring and Spatial Localisation for Sow Oestrus Detection. Animals.

[21] Lei K., Li B., Zhong S., Yang H., Wang H., Tang X., Xiong B. (2025). Research on video behavior detection and analysis model for sow estrus cycle based on deep learning. Agriculture.

[22] Sankarganesh D., Krishna Prasad P.D., Sujit S., Gnana Thiraviam A.I., Ambrose D.J. (2026). Estrus detection in dairy cattle: An updated review on strategies and technologies. Front. Vet. Sci..

[23] Bruinjé T., Morrison E., Ribeiro E., Renaud D., Serrenho R.C., LeBlanc S.J. (2023). Postpartum health is associated with detection of estrus by activity monitors and reproductive performance in dairy cows. J. Dairy Sci..

[24] Liao R., Xiao C., Lv Y., Liu Y., Lin Y., Zhu L. (2025). Whole-Plant Rape Silage-Based Diets for Chongming white goats: An Integrated Assessment of Growth Performance, Meat Quality and Gut Microbiota. Foods.

[25] Lin Y., Sun L., Dai J., Lv Y., Liao R., Shen X., Gao J. (2024). Characterization and Comparative Analysis of Whole-Transcriptome Sequencing in High- and Low-Fecundity Chongming White Goat Ovaries during the Estrus Phase. Animals.

[26] Lei K., Li B., Yang H., Wang H., Wang D., Xiong B. (2025). Spatiotemporal Modeling and Intelligent Recognition of Sow Estrus Behavior for Precision Livestock Farming. Animals.

[27] Lyu Z., Lu A., Ma Y.J.A.s. (2024). Improved YOLOv8-Seg based on multiscale feature fusion and deformable convolution for weed precision segmentation. Appl. Sci..

[28] Bai R., Wang M., Zhang Z., Lu J., Shen F. (2023). Automated construction site monitoring based on improved YOLOv8-seg instance segmentation algorithm. IEEE Access.

[29] Choi J.D., Kumar V.J.b. (2026). Adoption of MMPose, a general purpose pose estimation library, for animal tracking. bioRxiv.

[30] Sengupta A., Jin F., Zhang R., Cao S. (2020). mm-Pose: Real-time human skeletal posture estimation using mmWave radars and CNNs. IEEE Sens. J..

[31] Dolecheck K.A., Silvia W.J., Heersche G., Chang Y.M., Ray D.L., Stone A.E., Wadsworth B.A., Bewley J.M. (2015). Behavioral and physiological changes around estrus events identified using multiple automated monitoring technologies. J. Dairy Sci..

[32] Lodkaew T., Pasupa K., Loo C.K. (2023). CowXNet: An automated cow estrus detection system. Expert Syst. Appl..

[33] Medan M.S., Watanabe G., Sasaki K., Groome N.P., Sharawy S., Taya K. (2005). Follicular and hormonal dynamics during the estrous cycle in goats. J. Reprod. Dev..

[34] El-Tarabany M.S., El-Tarabany A.A., Atta M.A. (2019). Effect of season on hormonal profile and some biochemical parameters at different stages of estrous cycles in Baladi goats. Biol. Rhythm Res..

[35] Fatet A., Pellicer-Rubio M.T., Leboeuf B. (2011). Reproductive cycle of goats. Anim. Reprod. Sci..

[36] Ghosh S., Singh A.K., Haldar C.J. (2013). Adaptive and ecological significance of the seasonal changes in hematological, biochemical and hormonal parameters in the tropical goat (*Capra hircus*). J. Endocrinol. Reprod..

[37] Barkawi A., Elsayed E.H., Ashour G., Shehata E. (2006). Seasonal changes in semen characteristics, hormonal profiles and testicular activity in Zaraibi goats. Small Rumin. Res..

