# Supplementary material for: Toward Vision-Based Estrus Monitoring in Chongming White Goats: Behavioral Manifestations and Serum Hormone Correlations
Source: Animals (Basel). 2026 Jul 12;16(14):2161. doi: 10.3390/ani16142161 (PMC13404586; doi:10.3390/ani16142161)
Supplement: Supplementary file 1 [file animals-16-02161-s001.zip › animals-4410599-supplementary.pdf]

Table S1. Feed composition and nutrient levels of Chongming white goat diets (air-dry basis).

Items	Content
**Ingredients (%)**	
Corn	15
Soybean meal	12
Wheat bran	4
Corn Straw Silage	66.2
NaCl	1.3
Premix [1]	1.5
Total	100
**Nutrient levels**	
Crude protein (%)	8.1
Acidity detergent fiber (ADF) (%)	22.68
Neutral detergent fiber (NDF) (%)	34.36
Calcium (%)	0.93
Phosphorus (%)	0.90

[1]The premix provides the following per kg of diets: vitamin A 16,000 IU, vitamin D 5,000 IU, vitamin E 600 IU, Fe (as ferrous sulfate) 330 mg, Zn (as zinc sulfate) 260 mg, Cu (as copper sulfate) 60 mg, Mn (as manganese sulfate) 50 mg, I (as potassium iodide) 56 mg, Co 2.25 mg.
